# Network Pharmacology-Based Investigation of the Molecular Mechanisms of the Chinese Herbal Formula Shenyi in the Treatment of Diabetic Nephropathy

**DOI:** 10.3389/fmed.2022.898624

**Published:** 2022-06-10

**Authors:** Keng Chen, Yiyao Deng, Shunlai Shang, Ping Li, Linchang Liu, Xiangmei Chen

**Affiliations:** ^1^Clinical Medical School, Guangdong Pharmaceutical University, Guangzhou, China; ^2^First Medical Center of Chinese PLA General Hospital, Nephrology Institute of the Chinese People's Liberation Army, State Key Laboratory of Kidney Diseases, National Clinical Research Center for Kidney Diseases, Beijing Key Laboratory of Kidney Disease Research, Beijing, China; ^3^Department of Nephrology, Guizhou Provincial People's Hospital, Guiyang, China; ^4^Department of Nephrology, Beijing Hospital of Integrated Traditional Chinese and Western Medicine, Beijing, China

**Keywords:** Shenyi, network pharmacology, diabetic nephropathy, Chinese herbal compound, formula

## Abstract

**Background:**

The Chinese herbal formula Shenyi (SY) is a prescription that was developed by the Department of Nephrology, Chinese People's Liberation Army General Hospital. This preparation is mainly used to treat chronic kidney disease (CKD) caused by Diabetic nephropathy (DN) and is effective. However, the active ingredients of SY, DN treatment-related molecular targets and the effector mechanisms are still unclear.

**Methods:**

The Traditional Chinese Medicine Systems Pharmacology (TCMSP) database and the Traditional Chinese Medicine and Chemical Component Database of Shanghai Institute of Organic Chemistry were used to screen the active ingredients in SY, the TCMSP database and Swiss Target Prediction database were used to collect the targets of the active ingredients of SY, and the Gene Cards and Online Mendelian Inheritance in Man (OMIM) databases were used to screen for DN pathogenesis targets. The intersections of the component targets and disease targets were mapped to obtain the therapeutic targets. The METASCAPE database was used to perform Gene Ontology (GO) and Kyoto Encyclopedia of Genes and Genomes (KEGG) enrichment analyses of the therapeutic targets. Cytoscape 3.7.2 was used to analyze topological parameters and construct a network of SY for the treatment of DN.

**Results:**

Sixty-two active ingredients and 497 active ingredient effector targets in SY, 3260 DN-related targets, and 271 SY treatments for DN targets were identified. Among these targets, 17 were core targets, including AKT1, tumor necrosis factor (TNF), interleukin-6 (IL6), and TP53. The GO and KEGG enrichment analyses show that SY's therapeutic effects for DN occur mainly through pathways such as advanced glycation end product (AGE)-RAGE, PI3K-Akt, and IL-17.

**Conclusion:**

Multiple active ingredients in SY exhibit treatment effects on DN by affecting metabolism, inhibiting inflammation, and affecting cell structure growth.

## Introduction

Diabetic nephropathy (DN) is the main cause of chronic kidney disease (CKD) and end-stage renal disease (ESRD) worldwide (1). DN is also one of the major causes of death among diabetes mellitus (DM) patients. In the US, approximately 200,000 DN patients receive ESRD treatment, and this number is increasing at a rate of 500,00 patients per year (1). In China, DN accounts for a high proportion of CKD cases, and statistical data show that since 2011, DN has overtaken glomerulonephritis as the main cause of CKD (2). Modern medical treatments for DN generally involve blood glucose and blood pressure control and a reduced-fat, limited-protein diet, while renal replacement therapy and organ transplantation are the main treatments for ESRD. Although new therapeutic strategies for DN have emerged in recent years, no single treatment can reverse or slow its progression (3). Traditional Chinese medicine is known for its low toxicity, high efficacy, and multiple targets. Many clinical studies have reported that traditional Chinese medicine can effectively improve the major symptoms of DN and delay its progression.

**Figure 1:**
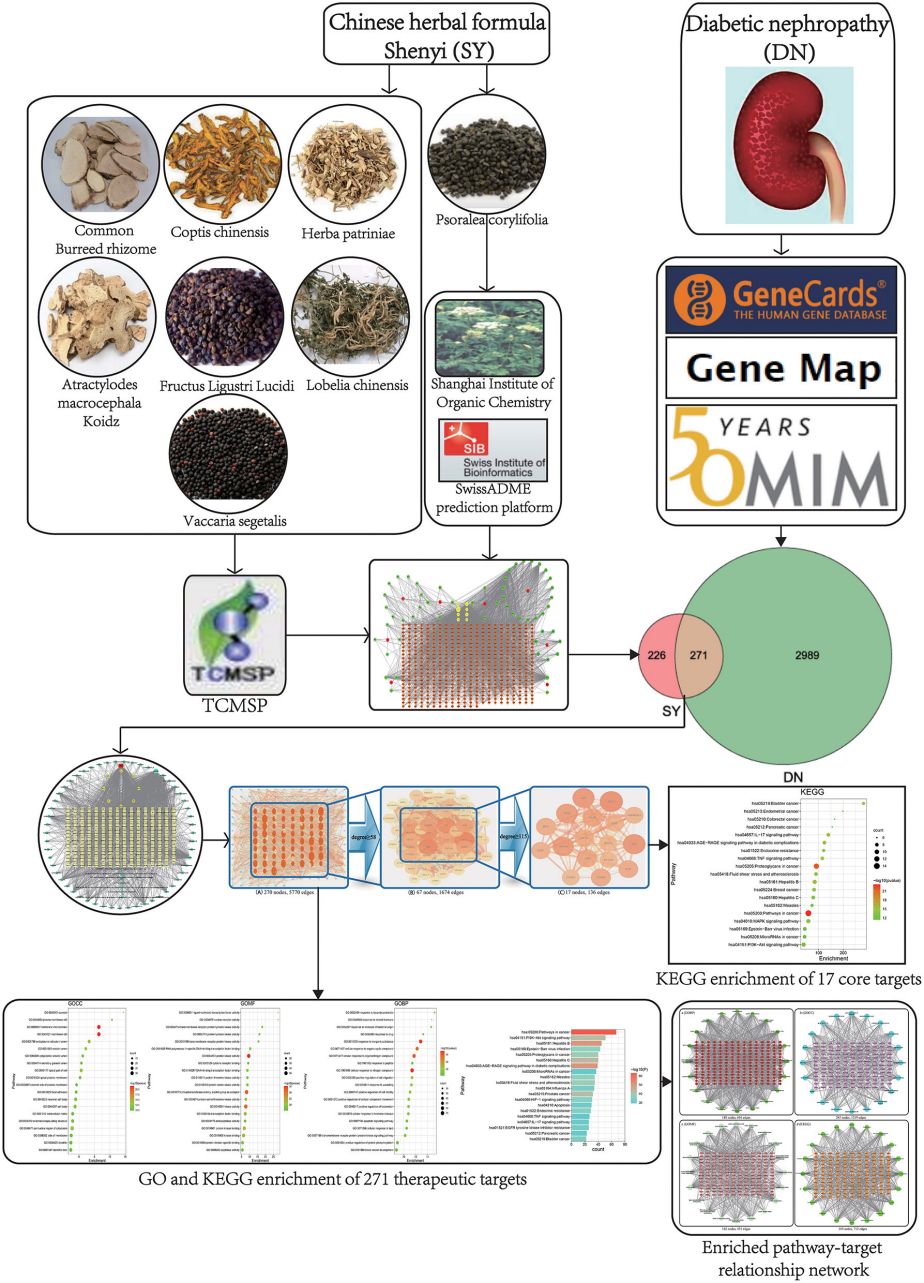
A flow chart exploring SY against DN based on network pharmacology.

The Chinese herbal formula Shenyi (SY) is a prescription that was developed by the Department of Nephrology, Chinese People's Liberation Army General Hospital. It consists of *Atractylodes macrocephala* Koidz, Herba patriniae, *Lobelia chinensis, Psoralea corylifolia, Coptis chinensis, Fructus Ligustri Lucidi*, common burreed rhizome, and *Vaccaria segetalis*. This preparation is mainly used to treat CKD caused by DN and the effect is good. Related studies have shown that SY can inhibit glomerular sclerosis by inhibiting the expression of glomerular TGF-β (4). The specific molecular targets and mechanisms of SY in DN are unknown. The traditional drug research method is not suitable for studies of traditional Chinese medicine formula with complex components and many targets. Traditional Chinese medicine network pharmacology is a novel research technique for studying active ingredients and predicting effector targets and is widely used to examine the active ingredient composition and effector mechanisms of traditional Chinese medicine and preparations and to determine their safety (5). In this paper, network pharmacology was employed as a research technique to examine the active ingredients of SY and the DN treatment-related molecular targets and effector mechanisms. A workflow chart is shown in Figure 1.

## Data And Methods

### Screening of the Active Ingredients in SY

*Atractylodes macrocephala* Koidz,” “Herba patriniae,” “*Lobelia chinensis,” “Coptis chinensis*,” “*Fructus Ligustri Lucidi*,” “common Burreed rhizome,” and “*Vaccaria segetalis*” were used as keywords to search in the Traditional Chinese Medicine Systems Pharmacology (TCMSP) database (6), and active ingredients with high activity were further selected based on an oral bioavailability (OB) ≥30% and a druglikeness (DL) ≥0.18. As “Psoralea corylifolia” was not included in the TCMSP database, it was used as a keyword in a search of the Traditional Chinese Medicine and Chemical Component Database of the Shanghai Institute of Organic Chemistry (7). Thus the active ingredients contained in *Psoralea corylifolia* were obtained. The PUBCHEM database (8) was used to obtain the molecular formula of the active ingredients of psoralen. And SwissADME prediction platform (9) were used to screen for active ingredients with high activity in *Psoralea corylifolia*.

### Prediction of Targets Corresponding to the Active Ingredients of SY and Network Construction

The targets of the active ingredients in *Atractylodes macrocephala* Koidz, Herba patriniae, *Lobelia chinensis, Coptis chinensis, Fructus Ligustri Lucidi*, common Burreed rhizome, and *Vaccaria segetalis* were obtained from the TCMSP database (8), and the SwissTargetPrediction system (10) was used to predict the targets of the active ingredients in *Psoralea corylifolia*. Cytoscape 3.7.2 was used to construct a drug-active ingredient-target network.

### Screening of DN-Related Targets and Prediction of the DN Targets of SY's Active Ingredients

“Diabetic nephropathy” was used as a keyword to search the GeneCards (11), Online Mendelian Inheritance in Man (OMIM) (12), and Gene Map databases to obtain DN-related disease targets. The aforementioned SY active ingredient targets and DN-related targets were mapped to obtain the DN targets of SY's active ingredients. A Venn diagram was plotted.

### Construction of the Drug-Active Ingredient-Therapeutic Target-Disease Network

The aforementioned therapeutic targets and SY's active ingredients were mapped. The therapeutic targets and the disease targets were then mapped, and Cytoscape 3.7.2 was used to construct a drug-active ingredient-therapeutic target-disease network.

### Construction of the Predicted SY Treatment-DN Target Protein-Protein Interaction (PPI) Network and Screening of Core Targets

The therapeutic targets were imported into the STRING PPI prediction platform (13), and the species was set as human, to construct the therapeutic target protein PPI network. Cytoscape 3.7.2 was used to build a visual PPI network. The PPI network TSV file obtained was imported into Cytoscape 3.7.2 to construct a PPI network based on “degree” and “combined score”. “Degree” was used as the limiting condition to screen for core targets of SY as a treatment for DN.

### Gene Ontology (GO) and Kyoto Encyclopedia of Genes and Genomes (KEGG) Enrichment Analysis and Construction of a Network of Enriched Pathways and Related Targets

GO and KEGG enrichment analyses of the therapeutic targets were performed using the METASCAPE analysis platform (14). The 271 therapeutic targets obtained through screening were imported into the METASCAPE platform, and GOBP, GOCC, GOMF, and KEGG enrichment analyses were performed. Finally, KEGG enrichment analysis was performed for 17 core targets. The top 20 pathways with the highest enrichment were selected for analysis and display, and a bubble chart, bar chart, and pathway-target network map were plotted.

## Results

### Screening of the Active Ingredients of the Different Chinese Herbal Medicines in SY

Preliminary screening of the TCMSP database (8) and the Traditional Chinese Medicine and Chemical Component Database of the Shanghai Institute of Organic Chemistry (9) identified 93 active ingredients in SY, 7, 14, 17, 19, 14, 13, 5, and 4 of which were present in *Atractylodes macrocephala* Koidz, Herba patriniae, *Lobelia chinensis, Psoralea corylifolia, Coptis chinensis, Fructus Ligustri Lucidi*, common Burreed rhizome, and *Vaccaria segetalis*, respectively. A total of 8 common ingredients are present in Chinese herbal medicines, including luteolin, quercetin, beta-sitosterol, kaempferol, stigmasterol, acacetin, linarin, and isovitexin. After common ingredients were removed, 77 active ingredients were identified in SY. Tables 1, 2 show the basic information of the top 30 core active ingredients in SY.

**Table 1:** Basic information about the active ingredients of seven herbs in SY.

**MOL ID**	**Molecule name**	**OB(%)**	**DL**	**Structure**
MOL002907	CorchorosideA_qt	104.95	0.78	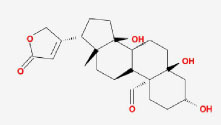
MOL005212	Olitoriside_qt	103.23	0.78	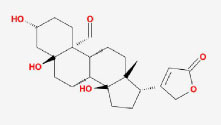
MOL009009	(+)-medioresinol	87.19	0.62	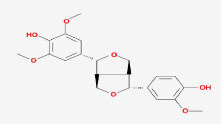
MOL008647	Moupinamide	86.71	0.26	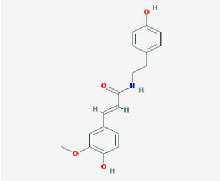
MOL005195	syringaresinoldiglucoside_qt	83.12	0.8	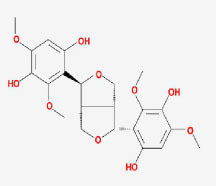
MOL012225	3-[(2S,3R)-2-(4-hydroxy-3-methoxy-phenyl)-3- (hydroxymethyl)-7-methoxy-2,3- dihydrobenzofuran-5-yl]propylacetate	73.08	0.54	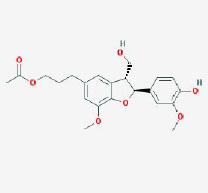
MOL005190	eriodictyol	71.79	0.24	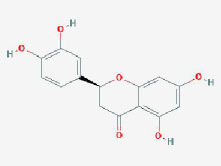
MOL002341	Hesperetin	70.31	0.27	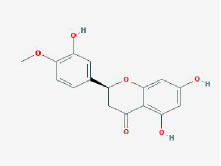
MOL000392	formononetin	69.67	0.21	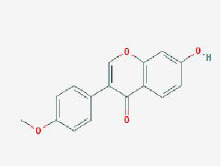
MOL005211	Olitoriside	65.45	0.23	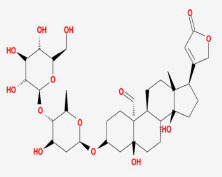
MOL000785	palmatine	64.6	0.65	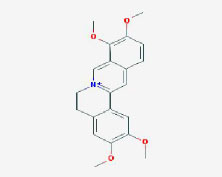
MOL012216	norlobelanine	64.08	0.3	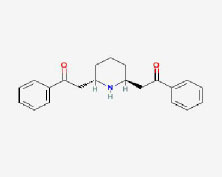
MOL000622	Magnograndiolide	63.71	0.19	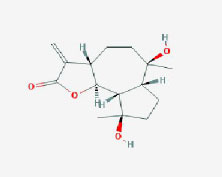
MOL001697	Sinoacutine	63.39	0.53	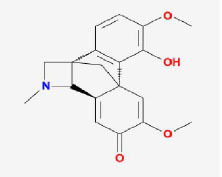
MOL000022	14-acetyl-12-senecioyl-2E,8Z,10E-atractylentriol	63.37	0.3	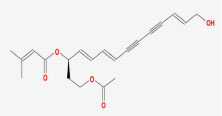
MOL000020	12-senecioyl-2E,8E,10E-atractylentriol	62.4	0.22	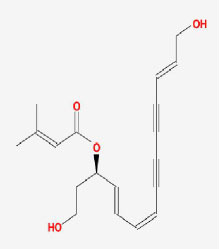
MOL012207	lobelanidine	60.53	0.32	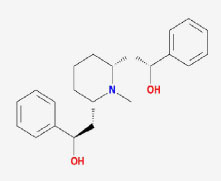
MOL000021	14-acetyl-12-senecioyl-2E,8E,10E-atractylentriol	60.31	0.31	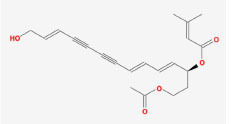
MOL001677	asperglaucide	58.02	0.52	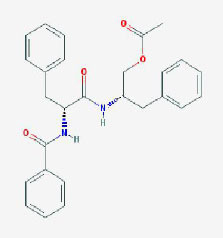
MOL004576	taxifolin	57.84	0.27	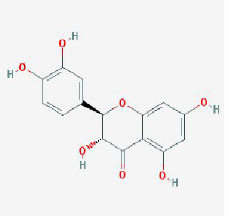

**Table 2:** Basic information on the active ingredients of *Psoralea corylifolia* in SY.

**CAS/PubChem CID**	**Molecule name**	**GI ABSORPTION**	**DRUGLIKENESS**					**Structure**
			**Lipinski**	**Ghose**	**Veber**	**Egan**	**Muegge**	
CAS: 63109-31-9	corylidin	High	yes	yes	yes	yes	yes	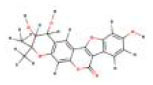
CAS: 298-81-7	8-methoxypsoralen	High	yes	yes	yes	yes	yes	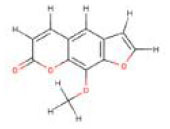
CAS: 24672-86-4	isobavachin	High	yes	yes	yes	yes	yes	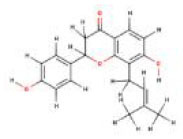
CAS: 3564-61-2	isopsoralidin	High	yes	yes	yes	yes	yes	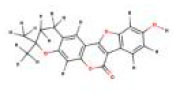
CAS: 76444-57-0	isoneobavachalcone	High	yes	yes	yes	yes	yes	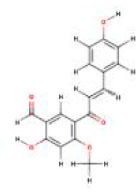
CAS: 19879-32-4	corylifolin bavachin	High	yes	yes	yes	yes	yes	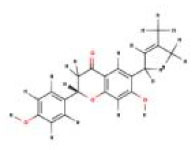
CAS: 19879-30-2	bavachinin	High	yes	yes	yes	yes	yes	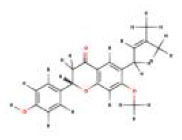
CAS: 74061-77-1	bavachromanol	High	yes	yes	yes	yes	yes	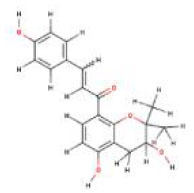
CAS: 41743-38-8	bavachromene	High	yes	yes	yes	yes	yes	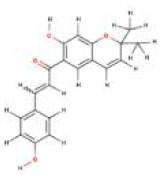
CAS: 53947-92-5	corylin	High	yes	yes	yes	yes	yes	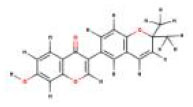

### Construction of the Drug-Active Ingredient-Target Network

**Figure 2:**
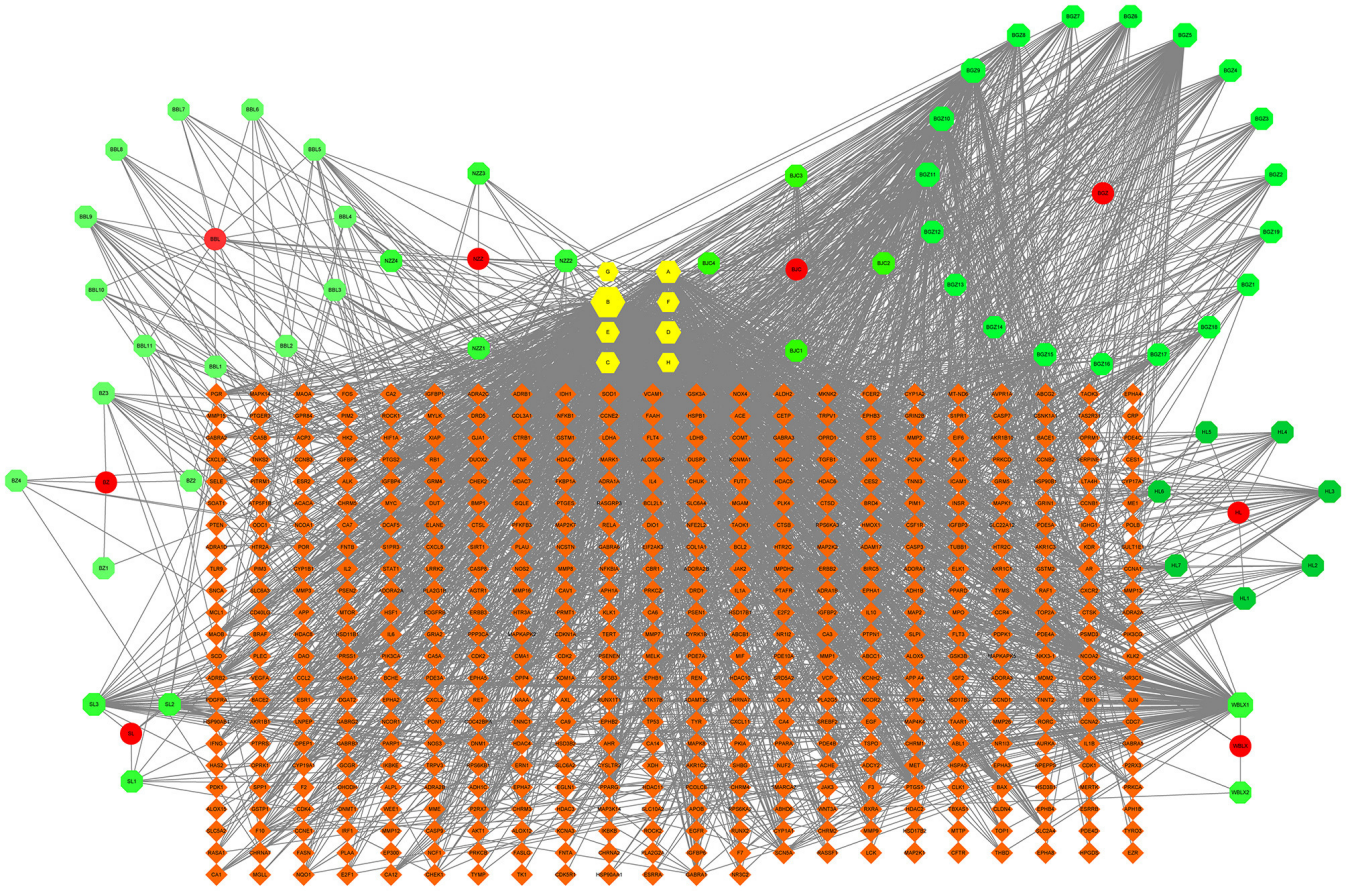
The drug-active ingredient-target network map for SY. In the figure, the green nodes represent active ingredients, the yellow nodes represent active ingredients common to 2 or more Chinese herbal medicines, the red nodes represent the 8 Chinese herbal medicine components of SY, and the orange nodes represent target genes. BZ represents *Atractylodes macrocephala* Koidz, andBZ1-4 represent the unique active ingredients of *Atractylodes macrocephala* Koidz; BZC represents Herba patriniae, and BZC1-4 represent the unique active ingredients of Herba patriniae; BBL represents *Lobelia chinensis*, and BBL1-11 represent the unique active ingredients of *Lobelia chinensis*; BGZ represents *Psoralea corylifolia*, and BGZ1-19 represent the unique active ingredients of *Psoralea corylifolia*; HL represents *Coptis chinensis*, and HL1-7 represent the unique active ingredients of *Coptis chinensis*; NZZ represents *Fructus Ligustri Lucidi*, and NZZ1-4 represent the unique active ingredients of *Fructus Ligustri Lucidi*; SL represents common Burreed rhizome, and SL1-3 represent the unique active ingredients of common Burreed rhizome; WBLX represents *Vaccaria segetalis*, and WBLX1 and 2 represent the unique active ingredients of *Vaccaria segetalis*. The degree of each node is reflected in its size.

After screening, 497 active ingredient-related targets of SY and 62 active ingredients were identified and used to construct the drug-active ingredient-target network (Figure 2), which contained 567 nodes and 2,523 edges, 8 of which were single traditional Chinese medicine nodes. Sixty-two active ingredient nodes and 497 target genes were unique to the drugs.

### Screening of DN-Related Targets

A total of 3,05,958 and 253 DN-related targets were obtained by screening the GeneCards (14), OMIM (12), and Gene Map databases, respectively. The targets obtained from the 3 databases were combined to obtain 3,260 DN-related targets.

### Screening of the DN Treatment Targets of the Active Ingredients of SY and Construction of the Therapeutic Target PPI Network

**Figure 3:**
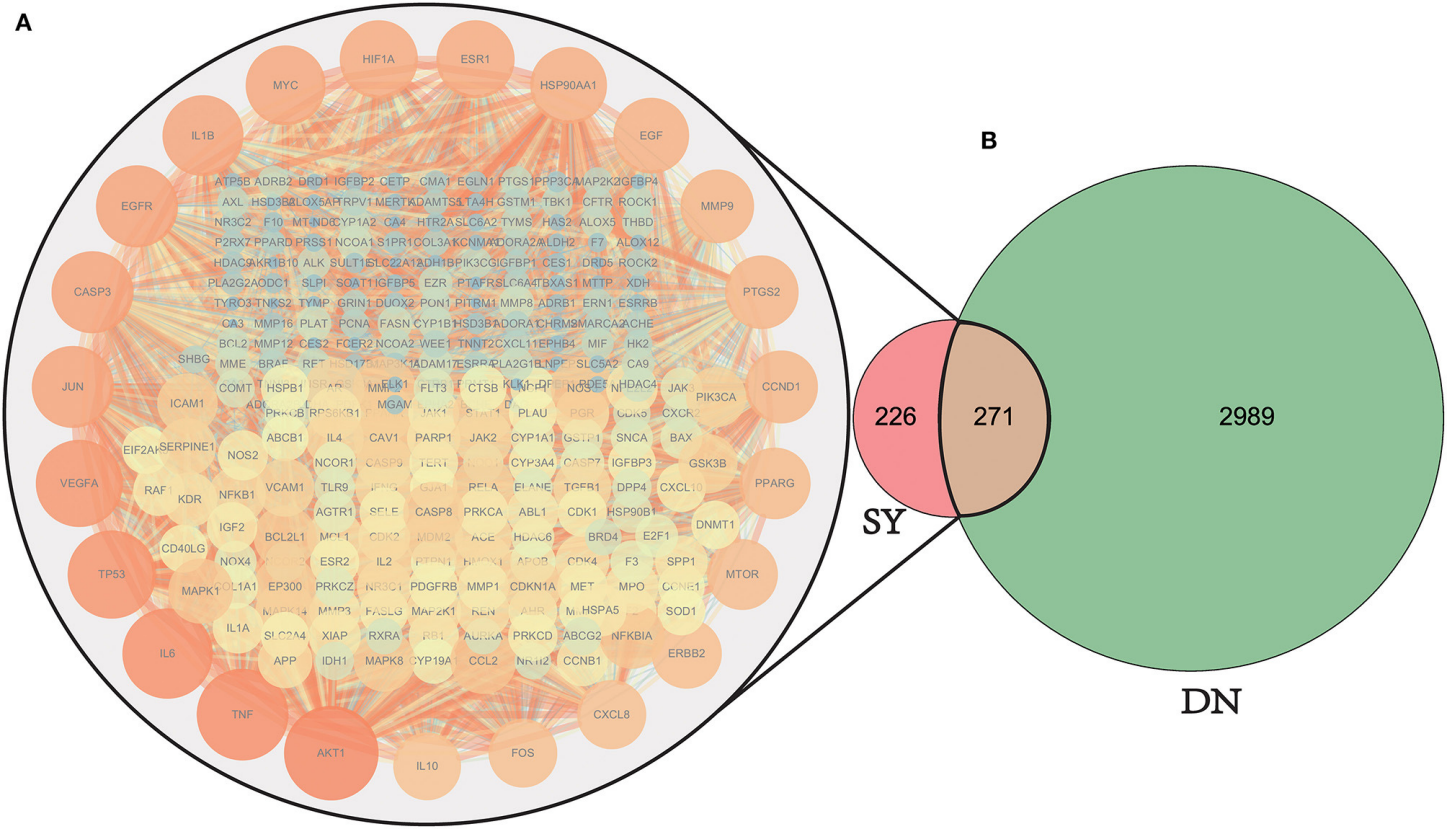
Venn diagram of drug target and disease target mapping and PPI map of the targets. DN, diabetic nephropathy; SY, Shenyi formula. A total of 3260 common DN-related targets, 497 common SY drug targets, and 270 intersecting targets were identified. (One of the targets with no PPI relevance was eliminated). **(A)** The PPI network map of the selected 270 SY-treated LN targets, the darker the color and the larger the size, the stronger the importance of the target. The targets on the outermost circle are the most central targets. **(B)** Venn diagram of SY therapeutic DN intersection targets.

The 497 SY active ingredient targets and 3,260 DN-related targets were mapped to obtain 271 DN treatment targets of the active ingredients of SY, and a Venn diagram was plotted (Figure 3B). The 271 therapeutic targets were imported into the STRING prediction platform to construct a PPI network (One of the targets with no PPI relevance was eliminated) (Figure 3A). After non-intersecting targets were removed, 271 nodes and 5,770 edges were obtained.

### Construction of the Drug-Active Ingredient-Therapeutic Target-Disease Network

**Figure 4:**
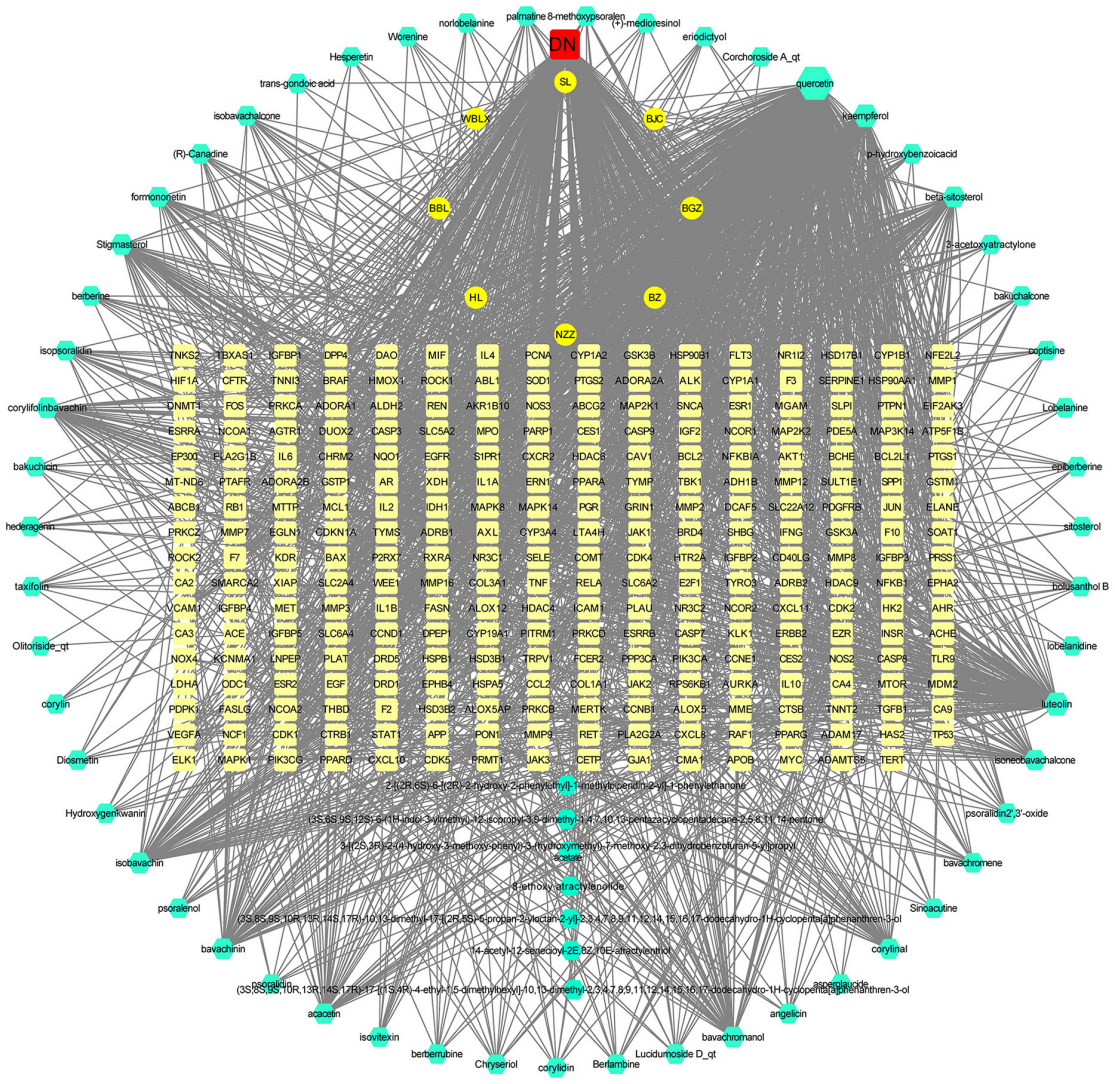
The drug-active ingredient-therapeutic target-disease network map. In the map, the green nodes represent the 8 Chinese herbal medicines in SY, the cyan nodes represent SY active ingredients, the pale yellow nodes represent effective drug treatment disease nodes, and the red nodes represent diabetic nephropathy. The corresponding degree of each node is reflected by its size.

The drug-active ingredient-therapeutic target-disease network (Figure 4) contained 342 nodes and 1,960 edges, 8 of which were Chinese herbal medicine nodes, 62 were active ingredient nodes, 271 were targets, and 1 was a disease.

### Construction of the PPI Map of the Predicted DN Targets of SY Treatment and the Screening of Core Targets

**Figure 5:**
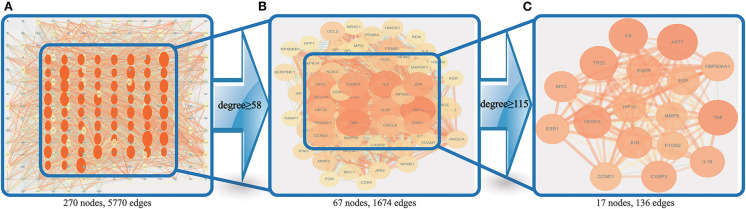
Core target screening flowchart. **(A)** shows the PPI network obtained by using importance ranking in Cytoscape; **(B)** shows the 67 nodes obtained via screening with a degree ≥58; and **(C)** shows the 17 nodes obtained via screening with a degree ≥115.

The PPI results of the 270 therapeutic target**s** were imported into Cytoscape 3.7.2 to obtain the PPI network (One of the targets with no PPI relevance was eliminated) (Figure 5A). Node size and color intensity were used to define the importance of the node: larger nodes, deeper color intensity, and more edges indicated greater importance. Values of 58 and 115 degrees were used as the lower limits to screen for core therapeutic targets in 2 runs. A total of 67 (Figure 5B) and 17 (Figure 5C) targets were obtained in the 1st and 2nd runs, respectively, and 17 core targets were finally obtained, namely, AKT1, TNF, IL6, TP53, VEGFA, EGFR, CASP3, JUN, IL1B, MYC, ESR1, HIF1A, HSP90AA1, EGF, PTGS2, MMP9, and CCND1. A core target screening flowchart was constructed.

### GO and KEGG Enrichment Analysis Results and the Pathway-Target Network

**Figure 6:**
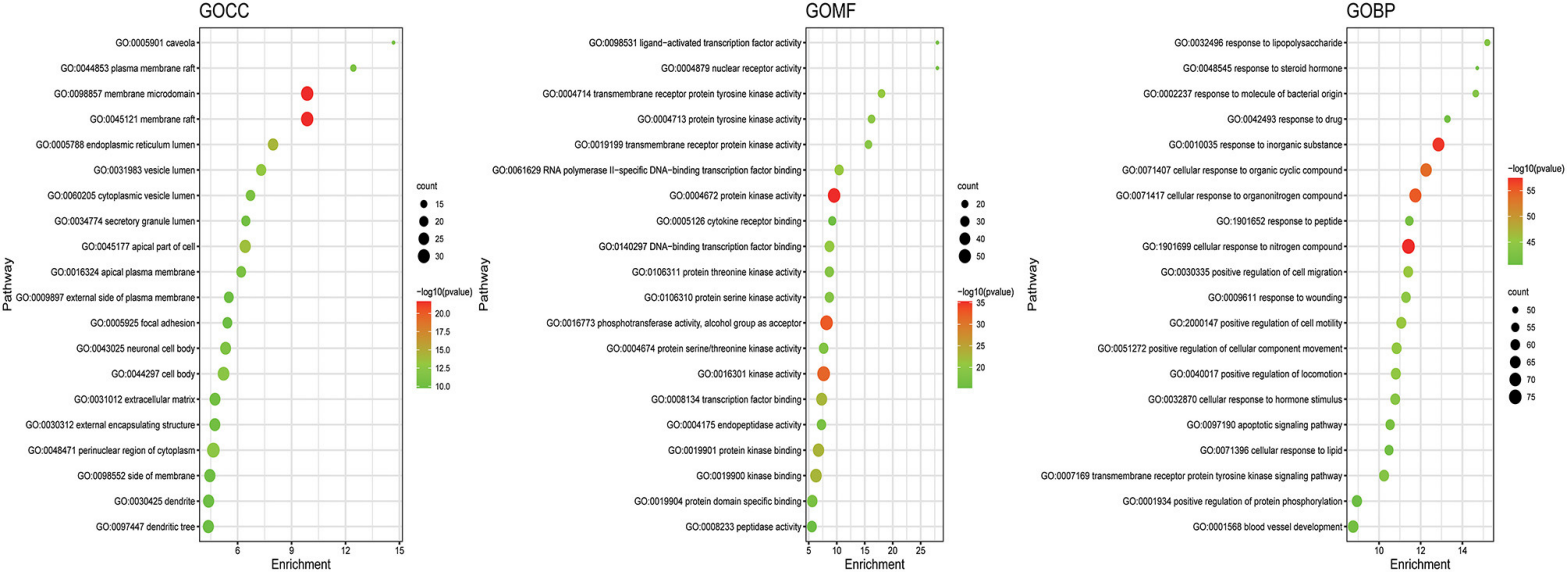
GO enrichment analysis of therapeutic targets. In the figure, the Y-axis coordinate, “pathway”, represents GO enrichment analysis pathways, the X-axis coordinate, “enrichment”, represents the degree of enrichment, and “count” represents the number of enriched targets in every pathway. A greater count indicates a higher degree of enrichment. The –log10 *P*-value is represented as a gradual color change from red to green. A higher -log10 *P* value corresponds to a lower *P* value, a greater degree of enrichment, and a redder color.

**Figure 7:**
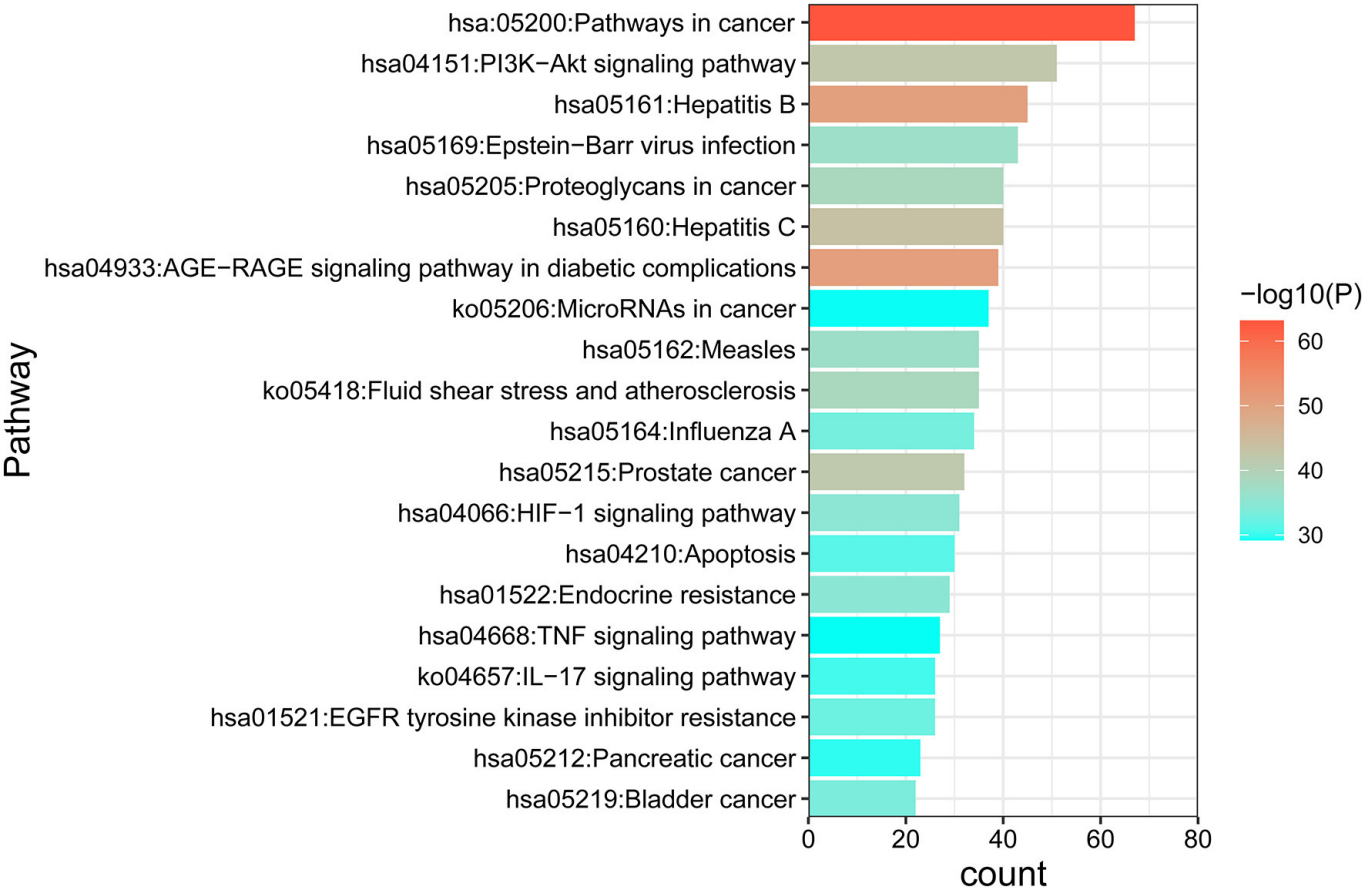
KEGG enrichment analysis of the DN treatment targets of SY. In the figure, the Y-axis coordinate, “pathway”, represents enriched pathways, and “count” represents the number of enriched targets in each pathway. The greater the count is, the higher the degree of enrichment. The *P*-value is represented as a gradual color change from red to blue. The higher the –log10 *P*-value is, the lower the *P*-value, the greater the degree of enrichment, and the redder the color.

**Figure 8:**
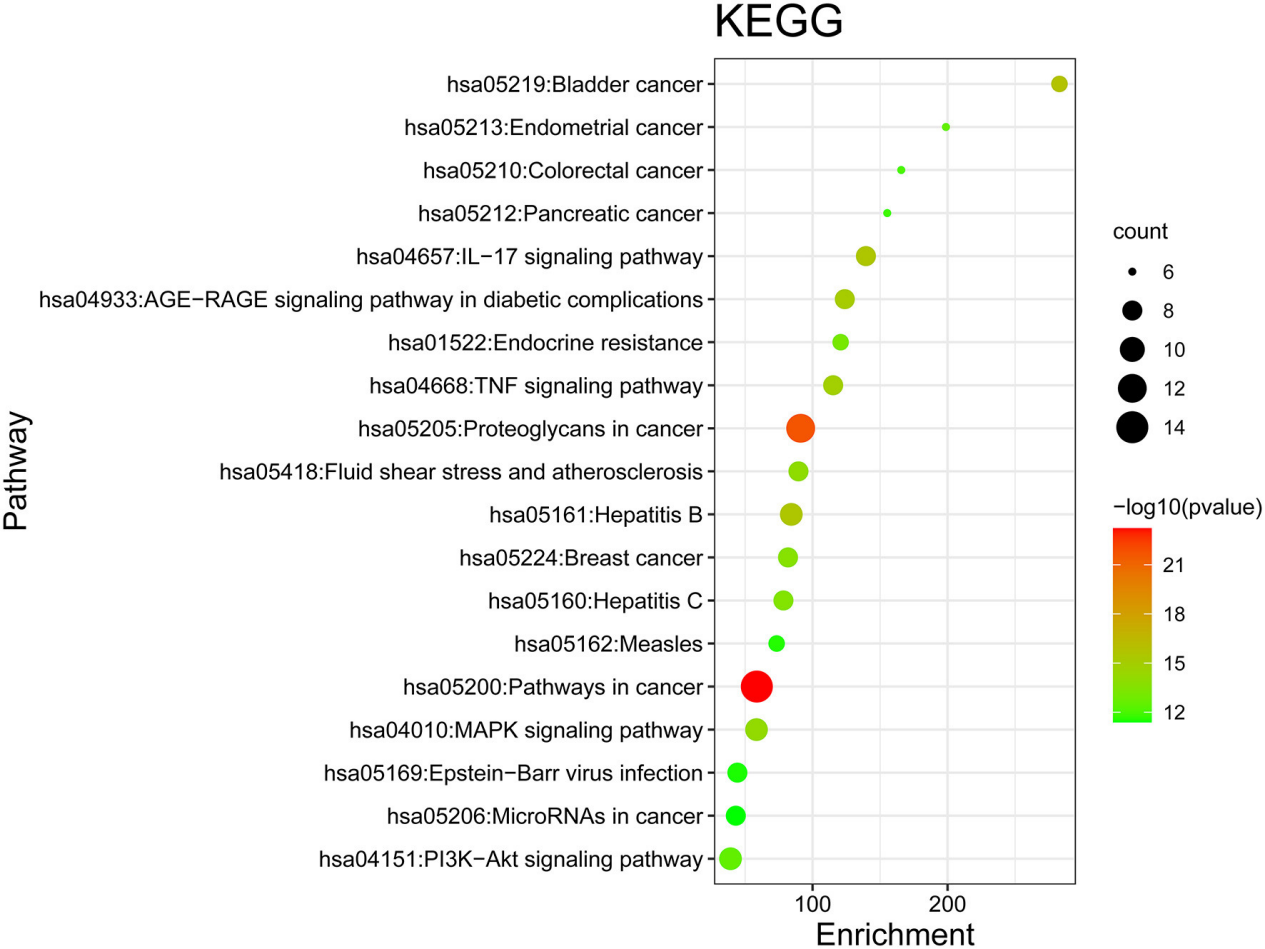
KEGG enrichment analysis of core targets. In the figure, the Y-axis coordinate, “pathway”, represents the GO enrichment analysis pathway, the X-axis coordinate, “gene ratio,” represents the degree of enrichment, and “count” represents the number of enriched targets in each pathway. The greater the count is, the higher the degree of enrichment. The –log10 *P*-value is represented as a gradual color change from red to green. The higher the –log10 *P*-value is, the lower the *P*-value, the greater the degree of enrichment, and the redder the color.

GO and KEGG enrichment analyses were performed on the 271 therapeutic targets to obtain 3,449 pathways (*P* < 0.01), of which 2,712, 128, 248, and 361 pathways were identified in GOBP, GOCC, GOMF, and KEGG, respectively (all *P* < 0.01). KEGG enrichment analysis was performed on the 17 core targets to obtain a total of 161 pathways (*P* < 0.01). A lower *P* value corresponded to a greater degree of enrichment. *P*-values were used for ranking to select the 20 pathways with the highest degree of enrichment for analysis (Figures 6, 7, 8). The KEGG enriched pathways were mainly in the advanced glycation end product (AGE)- RAGE signaling pathway, the PI3k-Akt signaling pathway, the hypoxia-inducible factor 1 (HIF-1) signaling pathway, and the endocrine resistance pathway in diabetic complications; the GOBP enriched pathways were mainly the cellular response to nitrogen compounds, the response to inorganic substances, the positive regulation of cell motility, the cellular response to hormone stimulus, and blood vessel development; the GOCC enriched pathways were mainly related to cellular microvesicular protein, cellular endoplasmic reticulum, vesicle lumens, dendrites, etc. The GOMF enriched pathways mainly involved protein kinase activity, protein kinase binding, transmembrane receptor protein tyrosine kinase activity, etc.

**Figure 9:**
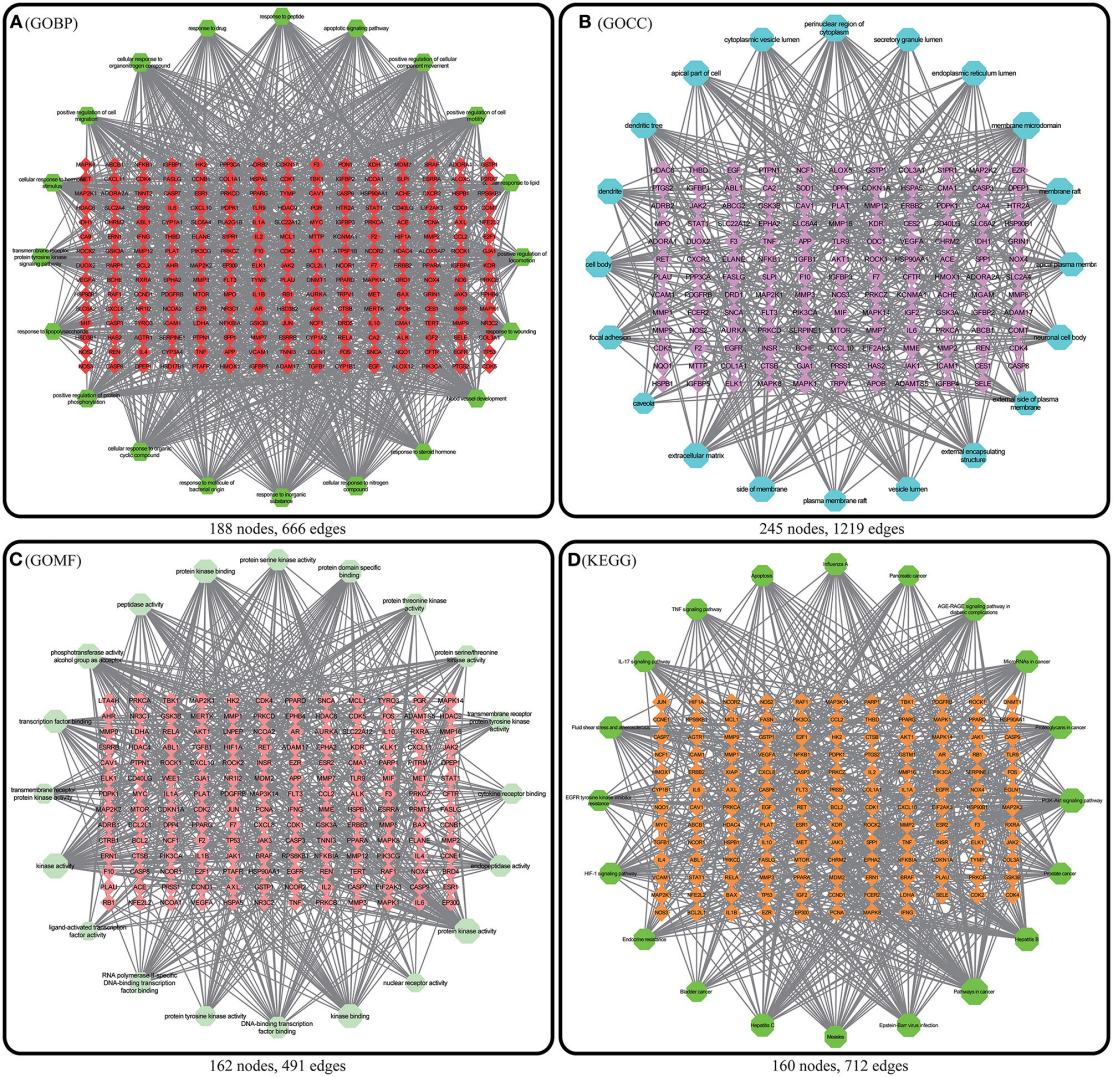
Enriched pathway-target relationship network. In the above 4 figures, the nodes with a matrix distribution in the central diamond represent pathway-related targets, and the nodes with a circular distribution in the peripheral hexagon represent pathways. The corresponding degree of each node is reflected by its size. **(A)** Enriched pathway-target relationship network of GOBP enrichment analysis. **(B)** Enriched pathway-target relationship network of GOCC enrichment analysis. **(C)** Enriched pathway-target relationship network of GOMF enrichment analysis. **(D)** Enriched pathway-target relationship network of KEGG enrichment analysis.

These enriched pathways were mapped to targets in the pathways to construct the interaction network of the GOBP (Figure 9A), GOCC (Figure 9B), GOMF (Figure 9C), and KEGG (Figure 9D) pathways and targets.

## Discussion

Network pharmacology analysis identified 77 active ingredients and 497 active ingredient effector targets of SY. When the active ingredients were sorted by degree, the active ingredients with a higher degree value included quercitrin, luteolin, kaempferol, beta-sitosterol, isobavachin, and bavachinin. Quercitrin is a flavonoid compound with diverse bioactivities that has antimicrobial, anti-inflammatory, anti-allergic, and blood pressure-lowering effects through multiple targets and pathways (15). Quercitrin is an active ingredient in many common Chinese herbal medicines, such as mugwort leaves, *Lobelia chinensis*, radix Bupleuri, Szechwan Chinaberry fruit*s*, and *Plantago asiatica* seeds. Related studies have reported that quercitrin can effectively improve streptozocin-induced DN in rats (16). Single-cell alkaline gel electrophoresis showed that quercitrin can alleviate oxidative stress in type 2 DM (17). Quercitrin can also provide effective protection against DN-related neuropathy (18). Related mechanistic studies have reported that the effects of quercitrin on DN are achieved by the inhibition of aerobic glycolysis through the HIF-1α/miR-210/ISCU/FeS pathway (19). The protective effects of quercitrin in diabetic neuropathy are achieved through inhibition of the Wnt/β-catenin signaling pathway (20). Luteolin is also an important active ingredient in SY treatment of DN, and luteolin is present in Herba patriniae, dense fruit dittany root bark, *Lobelia chinensis*, and *Mentha canadensis*. Luteolin has many pharmacological effects, such as anti-inflammatory, antiallergic, uric acid-lowering, antineoplastic, antimicrobial, and antiviral effects. Related studies showed that luteolin has important protective effects against nephrotoxicity in a long-term hyperglycemic rat model (21). A study found that luteolin can upregulate Nphs2 to significantly inhibit podocyte apoptosis, loss, and fusion in a diabetic rat model. Additionally, luteolin can inhibit glomerulosclerosis, maintain the normal physiological structure of the glomeruli, and delay the irreversible progression of DN (22). Kaempferol is an active flavonoid that is ubiquitous in *Lobelia chinensis*, Herba patriniae, Paeoniae radix alba, *Polygonum aviculare*, and other Chinese herbal medicines and has anti-inflammatory and antioxidant effects (23, 24). Recent studies have shown that kaempferol can promote the release of GLP-1 and insulin and inhibit RhoA/Rho kinase to ameliorate kidney injury and decrease fibrosis (25). Cellular experiments have shown that kaempferol can inhibit RhoA/Rho kinase activity in NRK-52E and RPTEC cells, thereby decreasing oxidative stress and inhibiting the expression of proinflammatory factors (TNFα and IL-1β) (26).

There were 271 predicted DN-related targets of SY treatment were identified. After KEGG and GO enrichment analyses were carried out on these 271 targets in the METACASE platform, the 20 pathways with the highest degree of enrichment were selected for analysis. Many of the top 20 pathways in the KEGG enrichment analysis were related to DN; the pathway with the highest degree of enrichment and a strong correlation with DN was the AGE-RAGE signaling pathway in diabetic complications. One study showed that AGE can bind to its receptor (RAGE) to induce oxidative stress and promote inflammation and thrombosis, thereby resulting in diabetes-related vascular complications. Furthermore, the formation and accumulation of AGEs can promote mitochondrial peroxide synthesis, resulting in further cytotoxicity and causing further kidney injury. In addition, this pathway and the renin–angiotensin system (RAS) can result in progression of diabetic vasculopathy (27). Hence, the therapeutic effect of SY on DN could result from its ability to block or inhibit interactions between the AGE pathway and other pathways. Another pathway that was enriched in KEGG was the PI3K-Akt signaling pathway, which has been shown to be related to diabetes. Akt can inhibit the expression of phosphoenolpyruvate carboxykinase and glucose 6-phosphatase to inhibit liver gluconeogenesis. Additionally, Akt can affect the translocation of glucose transporter type 4 (GLUT4) to regulate glucose uptake in muscle cells and adipocytes (28). Furthermore, AKT promotes the survival and proliferation of insulin-secreting β cells in the pancreas (29). Studies have found that Akt2 (R274H) mutation can cause severe hyperinsulinemia and DM in humans, which also shows that AKT actively participates in the *in vivo* regulation of metabolism (30). The therapeutic effects of SY on DN could result from its ability to positively regulate the Akt pathway. Many of the drugs in SY have anti-inflammatory effects, which is reflected in the interference in the IL-17 signaling pathway that was evident after KEGG enrichment. Th17 is a new type of effector CD4+ T-cell in the IL-17 signaling pathway that secretes IL-17A, which can promote the secretion of proinflammatory factors and macrophage infiltration, thereby exacerbating kidney damage in DN (31, 32). The enrichment of SY effector targets in this pathway shows that its therapeutic effects on DN may result from its inhibition of the effects of IL17A to alleviate kidney injury in DN. Among the top 20 pathways enriched in biological process (GOBP), SY's treatment effect on DN may be related to the pathways involved in the cellular response to nitrogen compounds, the cellular response to organonitrogen compounds, and the cellular response to organic cyclic compounds. Among the top 20 pathways enriched in cellular components (GOCC), SY's treatment effect on DN may be related to the pathways of the membrane microdomain, vesicle lumen, cellular microvesicular protein, and extracellular matrix. Among the top 20 pathways enriched in molecular function (GOMF), SY's treatment effects on DN may be related to the pathways of protein kinase activity, transcription factor binding, and protein tyrosine kinase activity.

KEGG enrichment analysis was carried out for the 17 core targets, and a high degree of overlap was noted between the results and the KEGG enrichment analysis of the initially selected 271 therapeutic targets, such as the PI3K-Akt signaling pathway, the AGE-RAGE signaling pathway in diabetic complications, and the IL-17 signaling pathway, showing the feasibility of employing the network pharmacology procedure used in this study to determine core therapeutic targets and pathways. The core target, AKT1, participates in metabolism, cell proliferation, cell survival, cell growth, and angiogenesis (33–36). TNF can induce insulin resistance and synergistically induce vascular endothelial growth factor (VEGF) production with IL1B and IL6 to affect angiogenesis (37). MYC is a transcription factor that binds DNA in a non-specific manner. MYC can bind to the promoter of VEGFA to promote VBEGFA production and angiogenesis (38). IL6, IL1B, and HSP90AA1 participate in inflammation and regulate many factors to induce inflammatory responses (39). Hence, SY exhibits treatment effects on DN treatment by affecting metabolism, inhibiting inflammation, and affecting cell structure growth.

## Conclusion

In this paper, network pharmacology was used to screen for targets related to SY treatment for DN, and gene enrichment analysis was used to predict the pathways involved in SY's treatment effects on DN. A literature search and analysis were performed on the active ingredients, therapeutic targets, and pathways of the Chinese herbal medicines contained in the formula, and the results indicated that SY has therapeutic effects on DN, which consistent with the results when SY is used in clinical practice. In summary, the treatment effect of SY on DN is the result of synergism among multiple components, multiple targets, and multiple pathways. Our study provides mechanistic evidence to supplemort SY as a treatment for DN in clinical practice and provides guidelines for subsequent SY and DN studies. The Chinese herbal formula Shenyi has been clinically used in the treatment of DN and has good curative effects. It can be developed as a drug for treating DN and preventing its progression to end-stage renal disease. The main purpose of this study is to predict the target pathway of compound Shenyi in the treatment of DN, and to provide a theoretical basis for better clinical development of drugs for the treatment of diabetic nephropathy. Since this research is based on the database mining research, the mechanism of drug action is only predicted, and no relevant experimental verification. Moreover, this paper uses multiple databases, and the algorithms of each database are different, which undoubtedly raises the question of the accuracy of network pharmacology analysis. In a follow-up study, we will carry out animal and cell-related experiments to verify the target pathway predicted in this study.

## Data Availability Statement

The datasets presented in this study can be found in online repositories. The names of the repository/repositories and accession number(s) can be found in the article/Supplementary Material.

## Author Contributions

KC carried out research design and implementation, research data collection and sorting, article conception and writing. YD carried out research planning and design and paper revision. SS and PL carried out some data sorting. XC and LL conducted research propose concepts and oversee the planning and execution of research activities. All authors contributed to the article and approved the submitted version.

## Funding

This work was supported by the National Natural Science Foundation of China (Grant No. 81830019) and the Beijing Natural Science Foundation (Grant No. 7202188).

## Conflict of Interest

The authors declare that the research was conducted in the absence of any commercial or financial relationships that could be construed as a potential conflict of interest.

## Publisher's Note

All claims expressed in this article are solely those of the authors and do not necessarily represent those of their affiliated organizations, or those of the publisher, the editors and the reviewers. Any product that may be evaluated in this article, or claim that may be made by its manufacturer, is not guaranteed or endorsed by the publisher.

## Supplementary Material

Supplementary Table S1 Information on active ingredients of 7 herbs (except Psoralea corylifolia) in SY.

Supplementary Table S2 Information on active ingredients of Psoralea corylifolia.

Supplementary Table S3 Disease targets obtained from GENECARD, OMIN and GENEMAP database.

Supplementary Table S4 Predicted targets of active ingredients in SY.

Supplementary Table S5 Predicted targets of SY in treatment of DN.

Supplementary Table S6 GOBP enrichment analysis pathway information of 271 targets for SY in treatment of DN.

Supplementary Table S7 GOCC enrichment analysis pathway information of 271 targets for SY in treatment of DN.

Supplementary Table S8 GOMF enrichment analysis pathway information of 271 targets for SY in treatment of DN.

Supplementary Table S9 KEGG enrichment analysis pathway information of 271 targets for SY in treatment of DN.

Supplementary Table S10 KEGG enrichment analysis pathway information of 17 core targets for SY in treatment of DN.
